# A synoptic overview of golden jackal parasites reveals high diversity of species

**DOI:** 10.1186/s13071-017-2329-8

**Published:** 2017-09-15

**Authors:** Călin Mircea Gherman, Andrei Daniel Mihalca

**Affiliations:** Department of Parasitology and Parasitic Diseases, Faculty of Veterinary Medicine, University of Agricultural Sciences and Veterinary Medicine Cluj-Napoca, Calea Mănăștur 3-5, 400372 Cluj-Napoca, Romania

**Keywords:** Golden jackal, *Canis aureus*, Parasites

## Abstract

The golden jackal (*Canis aureus*) is a species under significant and fast geographic expansion. Various parasites are known from golden jackals across their geographic range, and certain groups can be spread during their expansion, increasing the risk of cross-infection with other carnivores or even humans. The current list of the golden jackal parasites includes 194 species and was compiled on the basis of an extensive literature search published from historical times until April 2017, and is shown herein in synoptic tables followed by critical comments of the various findings. This large variety of parasites is related to the extensive geographic range, territorial mobility and a very unselective diet. The vast majority of these parasites are shared with domestic dogs or cats. The zoonotic potential is the most important aspect of species reported in the golden jackal, some of them, such as *Echinococcus* spp., hookworms, *Toxocara* spp., or *Trichinella* spp., having a great public health impact. Our review brings overwhelming evidence on the importance of *Canis aureus* as a wild reservoir of human and animal parasites.

## Background

The golden jackal, *Canis aureus* (Carnivora: Canidae) is a medium-sized canid species [1] also known as the common or Asiatic jackal [2], Eurasian golden jackal [3] or the reed wolf [4]. Traditionally, *Canis aureus* has been regarded as a polytypic species (Table 1), with 14 subspecies distributed across a vast geographical territory in Europe, Asia and Africa [5, 6]. Recently, phylogenetic studies have demonstrated that at least two of the African subspecies need a formal recognition as distinct species. Koepfli et al. [3] suggested that *C. aureus anthus* forms a distinct monophyletic lineage to *C. aureus* and should be recognized as a separate species. Similarly, the phylogenetic comparison of the Egyptian jackal (*C. aureus lupaster*) with other wolf-like canids showed a close relationship with the gray wolf species complex rather than with other subspecies of golden jackals [7]. Nevertheless, because most of the studies dealing with parasites of golden jackals do not mention the subspecies, for the purpose of this review we have considered the entire group, without excluding the two former subspecies.

**Table 1:** Subspecies and geographical distribution of the golden jackal, *Canis aureus*

Subspecies	Common name	Range	Synonyms
*Canis a. aureus* Linnaeus, 1758	Common jackal	Middle Asia; Afghanistan; Iran; Iraq; Arabian Peninsula; Baluchistan; northwestern India	*C. balcanicus*; *C. caucasica*; *C. dalmatinus*; *C. hadramauticus*; *C. hungaricus*; *C. kola*; *C. lanka*; *C. maroccanus*; *C. typicus*; *C. vulgaris*
*Canis a. algirensis* Wagner, 1841	Algerian wolf	Algeria; Morocco; Tunisia	*C. barbarus*; *C. grayi*; *C. tripolitanus*;
*Canis a. anthus* Cuvier, 1820	Senegalese wolf; grey jackal; slender jackal	Senegal	*C. senegalensis*
*Canis a. bea* Heller, 1914	Serengeti wolf; Serengeti jackal	Kenya; northern Tanzania	
*Canis a. cruesemanni* Matschie, 1900	Siamese jackal; South East Asian jackal	Thailand; Myanmar; East India	
*Canis a. ecsedensis* Kretzoi, 1947	Pannonian jackal	Pannonian Basin	*C. minor*; *C. balcanicus*; *C. hungaricus*
*Canis a. indicus* Hodgson, 1833	Indian jackal; Himalayan jackal	Pakistan; India; Nepal; Bhutan; Burma	
*Canis a. lupaster* Hemprich & Ehrenberg, 1833	African wolf; Egyptian wolf; Egyptian jackal	Egypt; Algeria; Mali; Ethiopian Highlands; Senegal	*C. lupaster*; *C. lupus lupaster*; *C. sacer*
*Canis a. moreoticus* Geoffroy Saint-Hilaire, 1835	European jackal; Caucasian jackal; Reed wolf	Southeastern Europe; Asia Minor; Caucasus	*C. graecus*
*Canis a. naria* Wroughton, 1916	Sri Lankan jackal	Southern India; Sri Lanka	*C. lanka*
*Canis a. palaestina* Khalaf, 2008		Palestine; Israel	
*Canis a. riparius* Hemprich & Ehrenberg, 1832	Somali wolf	Somalia; Ethiopia; Eritrea	*C. hagenbecki*; *C. mengesi*; *C. somalicus*
*Canis a. soudanicus* Thomas, 1903	Variegated wolf; Nubian wolf	Sudan; Somalia	*C. doederleini*; *C. nubianus*; *C. thooides*; *C. variegatus*
*Canis a. syriacus* Hemprich & Ehrenberg, 1833	Syrian jackal	Israel; Lebanon; Jordan	

The distribution of golden jackals is limited to the Old World [8]. Molecular evidence supports an African origin for all wolf-like canids including the golden jackal [8]. It is considered that the colonization of Europe by the golden jackal took place during the late Holocene and early Neolithic, through the Balkan Peninsula [9]. During the last century, the species has recorded at least two geographic expansion events. A notable expansion started in the 1950s, with a second one following during the 1980s. This is particularly evident in Europe. Stable reproductive populations have been recorded in about 20 European countries, while in other nine, vagrant specimens were observed [10]. The factors that facilitate the territorial expansion of golden jackals are unclear, but land use [11], climate change [12, 13], and the lack of intra-genus competition have been suggested [12–14].

Golden jackals have an opportunistic nutritional behaviour with an extremely varied diet [15]. They prey or scavenge on small mammals, birds and their eggs, amphibians, reptiles, even invertebrates, and they take carrion when available. Occasionally, jackals also feed on vegetables or fruits. Additionally, their relatively broad home range, varying from 1.1 to 20.0 km^2^ [16, 17], increases the chance of contact with various parasites but also with other hosts.

All these biological and behavioural features create premises for their infection with a broad range of pathogens, including parasites. Golden jackals are known to host a large spectrum of viral, bacterial and parasitic pathogens [18–20]. The literature survey indicates that the studies published on golden jackal parasites are usually limited to a country or, more commonly to a region, and there is no synoptic overview on this potentially important topic. The aim of the present work was to review all the published data on the parasite fauna of golden jackals in a comprehensive and updated list. The goal is consistent with the demographic and territorial expansion tendency of this species and increased contact with domestic animals and humans.

## Literature survey methodology

The list of the golden jackal parasites was compiled on the basis of an extensive literature search published from historical times until April 2017. Abstracts in conference proceedings and theses were also considered. The search queries were performed in the several databases: Pub Med [21], Science Direct [22], Web of Science [23], Helminthological Abstracts [24], Biological Abstracts [25], BioOne [26], Host-Parasite Database of the Natural History Museum (London) [27] and the web search engine Google Scholar [28]. Additionally, two Russian databases, namely the Russian Scientific Electronic Library [29] and the Scientific Library Earth Papers [30] were also used as sources of information.

The parasites are listed in tables, organized according to their taxonomic rank, and species within families are alphabetically listed. Taxonomy follows Adl et al. [31] for protists; Gibson et al. [32], Jones et al. [33], and Bray et al. [34] for trematodes; Kahlil et al. [35] and Nakao et al. [36] for cestodes; De Ley & Blaxter [37] for nematodes; Amin [38] for acanthocephalans; and the database “Catalogue of Life: 2016 Annual Checklist” by Roskov et al. [39] for arthropods. The names of the species were updated according to the current taxonomy, but synonyms used by different authors are also indicated. Each species is indexed together with the country of the report, the method of examination and reference. The records within a species are listed according to the alphabetical order of the country name. If two or more reports for the same country are registered, the ranking was made chronologically, according to the year publication. The prevalence, frequency and intensity of infection are also given, when available. The prevalence was provided or calculated only when the sample size was at least 10. In the case of experimental infection studies, the country has not been specified. Articles that report infections in captive jackals and doubtful records are mentioned and/or discussed accordingly.

## Protists

Eight families with 21 species were reported in golden jackals in 23 countries. Additionally, several protists were identified only to the generic level or were doubtfully considered as parasites of golden jackals (Table 2) [40–85].

**Table 2:** Protist parasites of the golden jackal, *Canis aureus*

Family	Species	Origin	Prevalence (%)	Frequency	Method	Reference
Phylum Apicomplexa						
Class Aconoidasida						
Babesiidae	*Babesia canis* (syn. *Piroplasma canis*)	na (as *P. canis*)	na	na	EI	[40]
		Nigeria^a^	na	1/6	BS	[41]
		Romania	9.2	5/54	MI	[42]
	*Babesia gibsoni*	na	na	na	EI	[43]
		India	na	na	BS	[44]
Theileriidae	“Theileria annae”	Romania	3.7	2/54	MI	[42]
Class Conoidasida						
Eimeriidae	*Eimeria* sp.^b^	Bulgaria	5.8	3/56	CO	[45]
	*Eimeria aurei* ^b^	India	na	na	CO	[46]
	*Isospora* sp.	Bulgaria	5.8	3/56	CO	[45]
		India^a^	case report		CO	[47]
		Iran	7.1	4/56	CO	[48]
		Serbia	6.6	4/60	CO	[49]
	*Isospora dutoiti*	former USSR	na	na	CO	[50]
		Turkmenistan	na	na	CO	[51]
	*Isospora kzilordiniensis*	Kazakhstan	na	2/9	CO	[52]
	*Isospora neorivolta*	Russia	9.3	14/150	CO	[53]
	*Isospora ohioensis*	Russia	5.3	8/150	CO	[53]
	*Isospora theileri*	Azerbaijan	na	na	CO	[54]
		Turkmenistan	na	na	CO	[51]
Hepatozoidae	*Hepatozoon* sp.	Algeria	na	2/5	MI	[55]
		Mauritania	25.0	4/16	MI	[55]
	*Hepatozoon canis*	Austria	case report		MI	[56]
			case report		MI	[42]
		Croatia	30.4	14/46	MI	[57]
		Czech Republic	na	1/1	MI	[42]
		Hungary	57.9	33/57	MI	[57]
			60.0	na	MI	[58]
		Israel	2.1	1/46	BS	[19]
		Montenegro	na	2/2	MI	[57]
		Romania	72.2	39/54	MI	[42]
		Serbia	67.5	140/206	MI	[57]
Sarcocystidae	*Cystoisospora canis*	Hungary	15.0	3/20	CO	[59]
	*Neospora caninum*	Israel	1.7	2/114	IFAT	[60]
	*Sarcocystis* sp.	Bulgaria	1.9	1/56	CO	[45]
	*Sarcocystis cruzi* (syn. *S. bovicanis*)	Russia (as *S. bovicanis*)	20.0	30/150	CO	[53]
	*Sarcocystis tenella* (syn. *S. ovicanis*)	Russia (as *S. ovicanis*)	34.0	51/150	CO	[53]
	*Sarcocystis tropicalis* (syn. *Isospora tropicalis*)	India (as *I. tropicalis*)	case report		CO	[61]
		India	case report		CO	[62]
	*Toxoplasma-*type oocysts	Hungary	5.0	1/20	CO	[59]
	*Toxoplasma gondii*	Iran	33.3	na	LAST	[63]
			77.5	31/40	ELISA	[64]
“Flagellates”^c^						
Hexamitidae	*Giardia* sp.	Iraq^a^	100	4/4	CO	[65]
	*Giardia duodenalis* (syn. *G. canis*)	Croatia	12.5	1/8	IF, MI	[66]
		Iran	7.1	4/56	CO	[48]
		Russia (as *G. canis*)	1.3	2/150	CO	[53]
Trichomonadidae	*Pentatrichomonas hominis* (syn. *P. canis auri*)	India (as *P. canis auri*)	case report		CO	[67]
Trypanosomatidae	*Leishmania* sp.	Iran	2.5	4/161	SIO	[68]
			12.5	6/48	IFA	
		Serbia	6.9	15/126	MI	[69]
		Spain^a^	case report		H	[70]
	*Leishmania donovani*	Bangladesh	5 cases	na	MI	[71]
		Georgia	na	1/4	SIO	[72]
		Kazakhstan	na	na	na	[73]
		Iran	5.0	1/20	SIO	[74]
		na	na	na	EI	[75]
	*Leishmania infantum*	Algeria	case report		IFI, MI	[76]
		Georgia	2.5	1/39	IA	[77]
		Iran	10.0	1/10	DAT, ELISA, IFAT	[78]
			11.6	7/60	DAT	[78]
			1.6	1/60	SIO	
		Iraq	59.6	90/151	SIO, Cult, IFAT, ELISA	[79]
		Israel	7.6	4/53	ELISA	[80]
			6.5	3/46	ELISA	[19]
			1.3	1/77	MI	[81]
		Kazakhstan	na	na	na	[82]
		Romania	2.7	1/36	MI	[42]
		Tajikistan	na	na	na	[83]
		Turkmenistan	2 specimens	na	na	[84]
	*Leishmania major*	na	na	na	EI	[85]
	*Leishmania tropica*	Israel	6.5	5/77	na	[81]

*Abbreviations: BS* blood smear May-Grünwald-Giemsa stained, *CO* coprological examination, *Cult* cultures from the viscera, blood and other tissues, *DAT* direct agglutination test, *EI* experimental infection, *ELISA* enzyme-linked immunosorbent assay, *H* histopathology, *IA* immunochromatographic assay, *IF* immunofluorescence assay, *IFAT* indirect fluorescent antibody test, *IFI* indirect fluorescent immunoassay, *LAST* latex agglutination slide test, *MI*- molecular identification, *SIO* smears from internal organs stained with standard Giemsa, *na* not applicable/unknown

^a^Animals kept in captivity

^b^Doubtful record

^c^Various opinions on the higher taxonomy of these groups are available, hence we keep the generic term “flagellates”

### *Leishmania*

The sand fly-borne kinetoplastids of the genus *Leishmania* were reported in golden jackals from 13 countries (Table 2), showing a large geographical distribution in Asia, Africa and Europe. At least three species of *Leishmania* have been identified by molecular methods in naturally infected golden jackals (*L. donovani*, *L. infantum* and *L. tropica*). Additionally, golden jackals were experimentally shown to be receptive for the infection with *L. major* [85], but this species has never been found in naturally infected specimens. The multiple records of *Leishmania* spp. in golden jackals suggest a reservoir role for this carnivore, for both visceral and cutaneous leishmaniasis in humans, as well as for canine leishmaniasis. Infected jackals have been found also at the margin of the endemic area for canine leishmaniasis (i.e. Romania), where this finding has been temporally correlated with the re-emergence of the disease in domestic dogs [86]. Although there is no clear link between the emergence of leishmaniasis in dogs and the spreading of jackals, this is an issue to be further investigated, mainly as the jackal continues to spread into areas at the margin of canine leishmaniasis endemicity. This was previously demonstrated when infected dogs were newly introduced to non-endemic areas in Europe [87].

### Tick-borne protists (Babesiidae, Theileriidae and Hepatozoidae)

Experimental evidence showed that golden jackals are receptive to the infection with *Babesia canis* [40] and *B. gibsoni* [43]. However, there are surprisingly few records of natural infections with piroplasms in golden jackals (Table 2) despite the large variety and number of studies on ticks (see below). In Europe, the only *Babesia* species molecularly confirmed in golden jackals is *B. canis*, recently reported in Romania [42]. The other report of *B. canis* in jackals is from Nigeria [41], but the species identification was based on blood smears and in captive animals. We consider this record doubtful, as the typical vector for *B. canis*, *Dermacentor reticulatus*, does not occur in Nigeria. Probably the species in this case belongs to the same complex group of large canine *Babesia* known in this area, *B. rossi* or *B. vogeli* [88]. *Babesia gibsoni*, which is widely distributed in Asia, has been reported only once in golden jackals, in India. Although *Babesia rossi* is common in domestic and wild carnivores in Africa [89], so far there are no records of this species in golden jackals. The scarcity of reports of *Babesia* spp. in this wild canid is probably related to the low number of studies and the lack of more sensitive/specific methods, as the typical vector ticks [*D. reticulatus* for *B. canis*, *Rhipicephalus sanguineus* (*sensu lato*) for *B. vogeli* and *Haemaphysalis leachi* for *B. rossi*] have been reported on various occasions on these hosts.

An interesting recent report indicates the presence of “Theileria annae” in golden jackals from Romania [42]. Currently, the taxonomic status of this species is debated and it is most commonly referred to as “*Babesia microti*-like”. This group has been reported predominantly in red foxes, but also in several other wild carnivores in North America, Asia and Europe [89]. However, so far, the role of golden jackals in its ecology remains unknown.

The first report of *Hepatozoon canis* in golden jackals is relatively recent [19] and has been followed in the last years by several records, mainly in Europe and North Africa (Table 2). Surprisingly, despite the wide distribution of *H. canis* in canids [90], this tick-borne apicomplexan has never been found in jackals from sub-Saharan Africa or Asia. Nevertheless, the large number of records and the presence of its main vector, *R. sanguineus* (*s.l*.) suggest a reservoir role of golden jackals for *H. canis* at least in Europe, Middle East and North Africa.

### Intestinal homoxenous coccidia (Eimeriidae)

Various species of intestinal coccidia of the family Eimeriidae have been found in, or even described from jackals (Table 2). We consider all records of *Eimeria* as pseudoparasites, as previously suggested [91]. Three species of the genus *Isospora* have been described from golden jackals but currently their taxonomic status is listed as doubtful [91]: *Isospora dutoiti* is a misidentification with *Hammondia* spp. or *Neospora caninum*, while *I. theileri* and *I. kzilordiniensis* are probably invalid names (as they might be synonyms with other *Isospora* species from canids). Two other species, *I. neorivolta* and *I. ohioensis*, which are known to infect several species of canids [91], were reported in golden jackals. Interestingly, all these *Isospora* reports in golden jackals are from countries in the former USSR, and this probably reflects a greater interest of researchers from this area for this group of parasites rather than the real geographical distribution. Few reports of unnamed *Isospora* sp. in golden jackals are known from Asia and the Balkans (Table 2).

### Heteroxenous coccidia (Sarcocystidae)

Various records list golden jackals host to Sarcocystidae. Sporocysts of *Sarcocystis* (*S. cruzi*, *S. tenella* and *S. tropicalis*) and oocysts of *Cystoisospora canis* have been reported in the faeces of golden jackals in Europe, Russia and India, suggesting their role as definitive hosts (Table 2). Although antibodies against *Neospora caninum* have been detected in *C. aureus* in Israel [60], the role of golden jackals as definitive hosts for this parasite has never been demonstrated and needs to be investigated. So far, various canid species were demonstrated to shed oocysts of *N. caninum*: dogs (*Canis familiaris*) [92], coyotes (*C. latrans*) [93], dingoes (*C. lupus dingo*) [94], and gray wolves (*C. lupus*) [95]. Interestingly, Takacs et al. [59] reported “*Toxoplasma*-like” oocysts in the faeces of jackals but, unfortunately, no morphometric data were provided and there was no attempt to characterize them molecularly. We can only assume that these were small oocysts which, in our opinion, could represent any of the small canine coccidia *N. caninum*, *Besnoitia* spp. or *Hammondia* spp., none of them confirmed so far in golden jackals.

## Helminths

The highest number of studies on the parasitic fauna of golden jackals are related to helminths. Our literature survey found at least 178 publications in 38 countries reporting helminths in golden jackals, with 119 species belonging to three phyla: Platyhelminthes, Nematoda and Acanthocephala [96–119].

### Trematodes

The diversity of trematodes in golden jackals is relatively high (27 species from nine families) (Table 3). Most of the studies originate in the countries of the former USSR, Asia and North Africa, with few scattered records in Europe. There are no trematodes recorded in golden jackals in sub-Saharan Africa. This situation reflects probably the impact of the Russian helminthological school and the lack of studies in other regions rather than the influence of ecological factors or feeding behaviour of jackals. Among the various records of trematodes in golden jackals, two groups could be identified: the canid- or Carnivora-specific trematodes and other trematodes (specific rather to other mammal groups or birds).

**Table 3:** A comprehensive list of trematode parasites of the golden jackal, *Canis aureus*

Family	Species	Origin	Prevalence (%)	Frequency	Intensity	Method	Reference
Phylum Platyhelminthes							
Class Trematoda							
Dicrocoeliidae	*Dicrocoelium dendriticum* (syn. *D. lanceatum*)	Russia	5.0	1/20	5.00 ± 4.45	necropsy	[96]^a^
			26.1	na	na	necropsy	[97]
			na	na	na	necropsy	[98]
Diplostomidae	*Alaria alata*	Azerbaijan	22.4	20/89	2–30	necropsy	[99]
			na	na	na	necropsy	[100]
		Bulgaria	9.0	na	na	necropsy	[101]
			1.9	1/56	na	necropsy	[45]
		Croatia	na	na	na	na	[102]
		Chechnya	100	16/16	22–268	necropsy	[103]
		Greece	20.0	1/5	na	necropsy	[104]
		Hungary	10.0	2/20	na	necropsy	[59]
		Iran	na	na	na	necropsy	[105]
		Russia	10.0	2/20	3.00 ± 2.68	necropsy	[96]
			34.8	na	na	necropsy	[97]
			na	na	na	necropsy	[98]
			13.3	8/60	na	necropsy	[106]
			na	na	na	necropsy	[107]
			26.0	39/150	2–23	necropsy	[53]
		Serbia	0.9	4/447	19.00 ± 3.63	necropsy	[108]
			30.0	18/60	na	necropsy	[49]
		Uzbekistan	na	na	na	na	[109]
	*Alaria americana* (syn. *A. canis*)^b^	Iran	5.0	1/20	34	necropsy	[110]
			10.0	1/10	na	necropsy	[111]
	*Pharyngostomum cordatum* (syn. *P. fausti*)	Azerbaijan	na	na	na	necropsy	[100]
		Azerbaijan (as *P. fausti*)	8.3	1/12	2	necropsy	[99]
			na	na	na	necropsy	[100]
		Russia	6.6	4/60	na	necropsy	[106]
Echinostomatidae	*Echinochasmus corvus*	India	na	na	na	na	[112]
	*Echinochasmus schwartzi*	Iran	na	na	na	necropsy	[105]
	*Euparyphium* sp.	Iran	na	na	na	necropsy	[105]
	*Euparyphium melis*	Russia	16.6	10/60	na	necropsy	[106]
Heterophyidae	*Ascocotyle italica* (syn. *Parascocotyle italica*)	Russia	8.3	5/60	na	necropsy	[106]
	*Ascocotyle sinoecum* (syn. *Phagicola sinoecum*)	Iran	na	na	na	necropsy	[105]
	*Cryptocotyle lingua* ^b^	Russia	2.7	4/150	3–18	necropsy	[53]
	*Heterophyes* sp.	Egypt	na	3/5	na	necropsy	[113]
	*Heterophyes aequalis*	Egypt	na	na	na	necropsy	[113]
	*Heterophyes dispar*	Egypt	na	na	na	necropsy	[113]
	*Heterophyes heterophyes*	Egypt	2 specimens	na	na	necropsy	[113]
	*Metagonimus ciureanus* (syn. *Dexiogonimus ciureanus*)	Georgia	na	na	na	necropsy	[114]
	*Metagonimus yokogawai*	Iran	14.2	4/28	na	necropsy	[115]
		Italy	case report		6	necropsy	[116]
		Serbia	1.6	1/60	na	necropsy	[49]
Microphallidae	*Microphallus narii* (syn. *Spelotrema narii*)	India	na	na	na	na	[117]
			na	na	na	na	[112]
Opisthorchiidae	*Metorchis xanthosomus* ^b^	Russia	21.8	na	na	necropsy	[97]
	*Opisthorchis* sp.	Bangladesh	na	1/5	na	necropsy	[118]
	*Pseudamphistomum truncatum*	Serbia	0.2	1/447	2	necropsy	[108]
			3.3	2/60	na	necropsy	[49]
Plagiorchiidae	*Plagiorchis* sp.	Iran	na	na	na	necropsy	[105]
	*Plagiorchis elegans* ^b^	Russia	3.3	2/60	na	necropsy	[106]
	*Plagiorchis massino*	Uzbekistan	na	na	na	na	[109]
Schistosomatidae	*Schistosoma spindale* ^b^	India	case report		na	CO	[119]
Troglotrematidae	*Nanophyetus salmincola* ^b^	India	case report		na	CO	[47]

*Abbreviations*: *na* not applicable/unknown, *CO* coprological examination

^a^Unknown site of infection

^b^Doubtful record

The most commonly reported and widely distributed trematode in golden jackals is *Alaria alata*, found in Caucasus, Russia and Central Asia to Middle East and the Balkans (Table 3). We consider the report of *Alaria americana* in Iran doubtful, as the species is known otherwise only in canids from North America [120].

Jackals have been commonly reported as hosts for fish-borne trematodes typically associated with carnivores. Such examples include species of the genera *Ascocotyle*, *Cryptocotyle*, *Heterophyes*, *Metagonimus* (Heterophyidae), *Echinochasmus*, *Euparyphium* (Echinostomatidae), *Pseudamphistomum*, *Opisthorchis* (Opisthorchiidae) mainly in Asia and northern Africa. The fish-borne *Nanophyetus salmincola* was identified in India, but its geographical distribution is limited to the Pacific Northwest of the USA [121]; with high probability, the report might represent a misidentification with *N. schikhobalowi*, an Asian troglotrematid [122]. The diversity of trematode species in golden jackals is completed by other groups which use various invertebrates (i.e. arthropods) (*Plagiorchis massino*, *Microphallus narii*) or non-fish small vertebrates (i.e. amphibians) (*Pharyngostomum cordatum*) as second intermediate hosts, reflecting the wide diet composition of this carnivore.

Interestingly, *Dicrocoelium dendriticum*, a hepatic fluke typically associated with herbivores, has been found on several occasions in the bile ducts of golden jackals [97, 98] in Russia. As the infection source for this parasite is represented by ant second intermediate hosts, the infection of jackals is probably accidental.

Several of these trematodes reported in golden jackals have zoonotic potential. Human alariosis caused by *Alaria* mesocercariae manifests in various clinical signs which range from cutaneous symptoms to respiratory disorders, a diffuse unilateral neuroretinitis even to an anaphylactic shock with fatal outcome [123]. However, all human cases originate in North America (and are probably caused by *A. americanum*). The zoonotic potential of *A. alata* in Eurasia remains unknown. Adults *Heterophyes dispar* and *H*. *heterophyes* may produce diarrhoea, abdominal pain and discomfort in humans [124], while *Metagonimus yokogawai* is considered to be the most common intestinal trematode infection in the Far East, highly important due to the ability of their eggs to invade the blood stream thus causing serious complications [125]. Hence, golden jackals might have a significant role in the environmental contamination with such parasites and represent an indirect source for human contamination. Hepatic and biliary trematodes *D*. *dendriticum*, *Pseudamphistomum truncatum* and *Opisthorchis felineus* are also able to infect humans, causing abdominal pain, weight loss, chronic relapsing watery diarrhea and hepatobiliary system damages [126, 127].

However, for many other trematode species, golden jackals, as other carnivores, are probably accidental hosts, or most likely, present a pseudoparasitism following ingestion of birds or rodents, as they typically infect other vertebrate groups. For instance, *Cryptocotyle lingua* is mainly a parasite of different gull species in Europe, North America and Japan [128]; *Plagiorchis elegans* is a parasite of raptors, waterfowl, passerines and several mammals as the wood mouse, rat, gerbil and hamster [129]; *Metorchis xanthosomus* is specific for birds in Anseriformes, Gaviiformes, Podicipediformes and Gruiformes [130]; and *Schistosoma spindale* has been described in ruminants and rodents in southeastern Asia [131].

### Cestodes

Cestode infections in golden jackals have been recorded across all their distribution range, with a relatively high species diversity (Table 4) [132–152].

**Table 4:** A comprehensive list of cestode parasites of the golden jackal, *Canis aureus*

Family	Species	Origin	Prevalence (%)	Frequency	Intensity	Method	Reference
Phylum Platyhelminthes							
Class Cestoda							
Dilepididae	*Aelurotaenia cameroni*	India	na	na	na	na	[132]
Diphyllobothriidae	*Diphyllobothrium* sp.	India^a^	na	na	na	CO	[133]
	*Diphyllobothrium latum*	Bangladesh	20.0	6/30	na	necropsy	[134]
		India	na	na	na	na	[112]
	*Spirometra* sp.	India^a^	na	na	na	CO	[135]
		Iran	7.1	1/14	4	necropsy	[136]
	*Spirometra erinaceieuropaei* (syn. *S. erinacei*)	Azerbaijan	25.0	19/76	1–19	necropsy	[99]
			na	na	na	necropsy	[100]
		Azerbaijan (as *S. erinacei*)	3.5	4/114	2–21	necropsy	[137]
		Iran (as *S. erinacei*)	na	na	na	necropsy	[105]
	*Spirometra houghtoni*	Iran	na	na	na	necropsy	[105]
	*Spirometra mansoni* (syn. *Bothriocephalus mansoni*)	Italy	case report	na	necropsy	[138]	
Dipylidiidae	*Diplopylidium noelleri*	Iran	5.0	na	na	necropsy	[139]
		Tunisia	16.0	5/31	1–66	necropsy	[140]
	*Dipylidium caninum* (syns *Taenia elliptica*, *T. cucumerina*)	Azerbaijan	na	na	na	necropsy	[100]
		Bangladesh	26.6	8/30	na	necropsy	[134]
		Bulgaria	3.8	na	na	necropsy	[141]
			63.6	7/11	na	necropsy	[142]
		Chechnya	100	16/16	3–12	necropsy	[103]
		Hungary	5.0	1/20	4	necropsy	[59]
		India	na	na	na	na	[143]
			na	na	na	na	[112]
		India^a^	5.0	3/60	na	CO	[144]
		Iran	7.1	1/14	4	necropsy	[136]
			10.0	4/40	na	necropsy	[145]
			20.0	2/10	na	necropsy	[111]
			10.1	8/79	na	necropsy	[139]
			33.9	19/56	na	necropsy	[48]
		Israel	46.6	7/15	na	necropsy	[146]
			5.8	1/17	na	CO	[19]
		Italy	na	na	na	necropsy	[138]
		Kazakhstan	16.6	3/18	2–8	necropsy	[147]
		Russia	47.8	na	na	necropsy	[97]
			5.0	1/20	1.00 ± 0.25	necropsy	[96]
			10.0	6/60	na	necropsy	[106]
			na	na	na	necropsy	[107]
			8.0	12/150	1–13	necropsy	[53]
		Serbia	1.6	7/447	4.8 ± 0.6	necropsy	[108]
		Tajikistan	na	na	na	necropsy	[148]
		Tunisia	na	1/5	na	necropsy	[149]
			16.0	5/31	4–67	necropsy	[140]
		Turkey	na	na	na	necropsy	[150]
		Uzbekistan	na	na	na	necropsy	[151]
			na	na	na	necropsy	[152]
	*Joyeuxiella echinorhynchoides*	Azerbaijan	30.2	23/76	1–30	necropsy	[99]
			na	na	na	necropsy	[100]
		Iran	27.8	5/18	na	necropsy	[334]
			7.5	3/40	na	necropsy	[145]
		Turkey	na	na	na	necropsy	[150]
	*Joyeuxiella pasqualei*	Iran	30.0	3/10	na	necropsy	[111]
Mesocestoididae	*Mesocestoides* sp. group A (oval to elongate cirrus-pouch and short cirrus)	Israel	8.1	7/86	na	necropsy, ME	[335]
	*Mesocestoides* sp. group B (broad-oval cirrus-pouch and long, more or less strongly coiled cirrus)	Israel	15.2	13/85	na	necropsy, ME	
	*Mesocestoides* sp.	Greece	na	3/5	na	necropsy	[104]
		Tunisia	na	2/5	na	necropsy	[149]
		Iran	na	1/1	na	necropsy	[336]
		Bulgaria	34.6	na	na	necropsy	[45]
	*Mesocestoides corti*	Azerbaijan	na	na	na	necropsy	[100]
		Tunisia	12.9	4/31	1–10	necropsy	[140]
	*Mesocestoides lineatus* (syn. *M. carnivoricolus*)	Azerbaijan	2.6	3/114	9–47	necropsy	[137]
			37.7	37/98	2–63	necropsy	[99]
			na	na	na	necropsy	[100]
		Bulgaria	27.0	na	na	necropsy	[101]
			72.7	8/11	na	necropsy	[142]
		Hungary	20.0	4/20	na	necropsy	[59]
		India	na	na	na	na	[112]
		India (as *M. carnivoricolus*)	na	na	na	necropsy	[337]
		Ingushetia	na	1/2	na	necropsy	[338]
		Iran	15.0	3/20	na	necropsy	[110]
			70.0	7/10	na	necropsy	[111]
			36.7	29/79	na	necropsy	[139]
			30.3	17/56	na	necropsy	[48]
			61.1	11/18	na	necropsy	[334]
		Russia	26.1	na	na	necropsy	[97]
			5.0	1/20	1.00 ± 0.53	necropsy	[96]
			40.0	24/60	na	necropsy	[106]
			na	na	na	necropsy	[339]
			na	na	na	necropsy	[98]
			40.0	60/150	1–128	necropsy	[53]
		Serbia	5.8	26/447	69.7 ± 9.3	necropsy	[108]
		Tajikistan	na	na	na	necropsy	[148]
		Tunisia	74.0	23/31	na	necropsy	[140]
		Turkey	na	na	na	necropsy	[340]
		Ukraine	na	1/1	5	necropsy	[341]
		Uzbekistan	na	na	na	necropsy	[151]
			na	na	na	necropsy	[152]
	*Mesocestoides litteratus*	Serbia	4.7	21/447	64.3 ± 15.1	necropsy	[108]
		Tunisia	23.0	7/31	6–130	necropsy	[140]
	*Mesocestoides petrowi*	Azerbaijan	na	na	na	necropsy	[100]
		Russia	na	na	na	necropsy	[342]
	*Mesocestoides zacharovae*	Azerbaijan	case report	na	necropsy	[343]	
Taeniidae	*Echinococcus granulosus*	Azerbaijan	16.3	16/98	2–400	necropsy	[99]
			na	na	na	necropsy	[100]
		Bangladesh	20.0	6/30	na	necropsy	[134]
		Bulgaria	23.0	na	na	necropsy	[101]
			na	3/3	na	PCR	[344]
			9.0	1/11	na	necropsy	[142]
			1.9	na	na	necropsy	[45]
		Ceylon	case report	7	necropsy	[345]	
		Chad	1.2	1/82	na	necropsy	[346]
		Chechnya	na	na	na	necropsy	[347]
			12.0	2/16	8–16	necropsy	[103]
		Chechnya, Ingushetia	na	2/7	74–217	necropsy	[348]
		Hungary	10.0	2/20	na	necropsy	[59]
		India	na	na	na	CO	[349]
		Iran	5.0	1/20	48	necropsy	[110]
			na	na	na	necropsy	[105]
			16.0	na	na	necropsy	[350]
			2.3	2/86	na	necropsy	[351]
			40.0	16/40	na	necropsy	[145]
			40.0	16/40	na	necropsy	[352]
			8.9	7/79	na	necropsy	[139]
			20.0	2/10	na	necropsy	[353]
			66.7	6/9	na	CO	[353]
			na	1/1	na	PCR	[354]
			3.5	2/56	na	necropsy	[48]
			na	na	na	PCR	[355]
		Italy	case report	1	necropsy	[356]	
		Kazakhstan	5.9	na	3–29	necropsy	[147]
		Kenya	27.2	6/22	< 200	necropsy	[357]
		Palestine	na	na	na	na	[358]
		Pakistan	9.0	9/100	na	necropsy	[359]
		Russia	12.5	2/16	na	necropsy	[360]
			82.6	na	na	necropsy	[97]
			69.8	na	na	necropsy	[361]
			5.0	1/20	4.00 ± 2.36	necropsy	[96]
			66.0	10/15	na	CO	[362]
			3.3	2/60	na	necropsy	[106]
			na	na	na	necropsy	[107]
		Tajikistan	30.7	4/13	> 1,000	necropsy	[363]
		Tunisia	na	1/5	72	necropsy	[149]
			na	2/2	na	PCR	[364]
			9.7	3/31	11–98	necropsy	[140]
	*E. multilocularis* (syn. *Alveococcus multilocularis*)	Azerbaijan	3.7	2/54	3–5	necropsy	[99]
		Hungary	9.0	1/11	412	necropsy	[365]
		Ingushetia	na	1/2	na	necropsy	[338]
		Iran	16.0	4/25	na	necropsy	[366]
			50.0	5/10	na	necropsy	[353]
			na	9/9	na	CO	[353]
		Russia (as *Alveococcus multilocularis*)	18.7	3/16	na	necropsy	[360]
		Serbia	14.3	4/28	4–57	necropsy	[367]
		Tajikistan	7.7	1/13	na	necropsy	[363]
		Uzbekistan	na	1/4	na	necropsy	[368]
	*Multiceps multiceps* (syn. *Taenia multiceps*	Azerbaijan	8.9	8/89	2–11	necropsy	[99]
			na	na	na	necropsy	[100]
		Bangladesh (as *T. multiceps*)	10.0	3/30	na	necropsy	[134]
		Bulgaria	9.0	na	na	necropsy	[101]
			9.0	1/11		necropsy	[142]
		Chechnya	na	na	na	necropsy	[347]
		Iran	7.5	3/40		necropsy	[145]
		India	na	na	na	necropsy	[369]
		Kazakhstan	11.1	2/18	4–16	necropsy	[147]
		Russia	39.1	na	na	necropsy	[97]
		Serbia	1.6	7/447	3.00 ± 0.53	necropsy	[108]
		Tajikistan	na	2/6	na	necropsy	[370]
		Ukraine	na	1/1	na	necropsy	[341]
	*Taenia serialis*	Kazakhstan	5.5	1/18	10	necropsy	[147]
		Kenya	na	2/2	42	PCR	[371]
		Serbia	1.1	5/447	2.7 ± 0.2	necropsy	[108]
	*Taenia* sp.	Bulgaria	23.0	na	na	necropsy	[141]
		Greece	na	1/5	na	necropsy	[104]
		India	na	na	na	na	[143]
		India^a^	11.6	7/60	na	CO	[144]
		Iran^a^	na	2/2	11 ± 2 epg	CO	[372]
		Kenya	60.0	3/5	na	necropsy	[373]
		Tunisia	19	6/31	1–29	necropsy	[140]
	*Taenia crassiceps*	Azerbaijan	na	na	na	necropsy	[100]
		Hungary	40.0	8/20	na	necropsy	[59]
		Russia	25.0	15/60	na	necropsy	[106]
	*Taenia hydatigena* (syn. *T. marginata*)	Azerbaijan	15.3	14/91	1–18	necropsy	[99]
			na	na	na	necropsy	[100]
		Bulgaria	55.0	na	na	necropsy	[101]
			27.2	3/11	na	necropsy	[142]
		Chechnya	na	na	na	necropsy	[347]
			50.0	8/16	3–6	necropsy	[103]
		Hungary	15.0	3/20	na	necropsy	[59]
		India	na	na	na	necropsy	[369]
		Iran	7.1	1/14	2	necropsy	[136]
			40.0	16/40	na	necropsy	[145]
			10.0	1/10	na	necropsy	[111]
			7.6	6/79	na	necropsy	[139]
			7.1	4/56	na	necropsy	[48]
			5.6	1/18	na	necropsy	[334]
		Italy (as *T. marginata*)	na	na	na	na	[138]
		Kazakhstan	22.0	4/18	2–8	necropsy	[147]
		Russia	6.2	1/16	na	necropsy	[360]
			36.8	14/38	1–3	necropsy	[374]
			34.8	na	na	necropsy	[97]
			5.0	1/20	3.00 ± 2.18	necropsy	[96]
			1.6	1/60	na	necropsy	[106]
			na	na	na	necropsy	[98]
			na	na	na	necropsy	[107]
		Serbia	0.9	4/447	3.75 ± 1.80	necropsy	[108]
		Tajikistan	na	na	na	necropsy	[148]
		Uzbekistan	na	na	na	necropsy	[152]
	*Taenia krabbei*	Azerbaijan	0.8	1/114	3	necropsy	[137]
			na	na	na	necropsy	[100]
	*Taenia krepkogorski* (syn. *Hydatigera krepkogorski*)	Azerbaijan	na	na	na	necropsy	[100]
	*Taenia ovis*	Azerbaijan	5.1	2/39	1–2	necropsy	[99]
			na	na	na	necropsy	[100]
		Bulgaria	case report		na	necropsy	[375]
		Iran	5.6	1/18		necropsy	[334]
		Russia	39.1	na	na	necropsy	[97]
		Tajikistan	na	na	na	necropsy	[148]
		USSR (former)	na	na	na	necropsy	[376]
	*Taenia pisiformis* (syn. *T. serrata*)	Azerbaijan	15.7	14/89	1–6	necropsy	[99]
			na	na	na	necropsy	[100]
		Bulgaria	18.0	na	na	necropsy	[101]
			54.5	6/11		necropsy	[142]
		Chechnya	100	16/16	3–18	necropsy	[103]
		Greece	na	1/5	na	necropsy	[104]
		Hungary	20.0	4/20	na	necropsy	[59]
		India (as *T. serrata*)	na	na	na	na	[377]
			na	na	na	necropsy	[369]
		Iran	na	na	na	necropsy	[105]
		Kazakhstan	5.5	1/18	3	necropsy	[147]
		Russia	17.4	na	na	necropsy	[97]
			3.3	2/60		necropsy	[106]
			na	na	na	necropsy	[107]
			16.7	25/150	1–3	necropsy	[53]
		Serbia	1.8	8/447	10.1 ± 2.1	necropsy	[108]
		Tajikistan	na	na	na	necropsy	[148]
		Tunisia	3.2	1/31	1	necropsy	[140]
	*Taenia polyacantha* (syn. *Tetratirotaenia polyacantha*)	Azerbaijan (as *Tetratirotaenia polyacantha*)	na	na	na	necropsy	[100]
		Turkey	na	na	na	necropsy	[340]
	*Taenia taeniaeformis* (syn. *Hydatigera taeneiformis*)	Azerbaijan (as *H. taeneiformis*)	na	na	na	necropsy	[100]
		Tajikistan	na	na	na	necropsy	[148]
		Uzbekistan	na	na	na	necropsy	[152]

*Abbreviations*: *CO* coprological examination; *ME* microscopic/morphological examination; *PCR* polymerase chain reaction; *epg* eggs per gram faeces; *na* not applicable/not available

^a^Animals kept in captivity

Among all the cestode species, *Aelurotaenia cameroni* is the only one known exclusively in the golden jackal. However, as the species was only recently described [132], its absence from other carnivores cannot be excluded until further studies. It is not surprising that all other identified tapeworm species are characteristic to carnivores, confirming the low specific affinity of the adult parasites [153]. As such species infect usually a wider range of canid or non-canid carnivores, this demonstrates a close environmental connection between multiple carnivore species and the use of the same trophic source.

The most commonly reported tapeworms in golden jackals are *Dipylidium caninum*, *Mesocestoides* spp., *Echinococcus granulosus* and *Taenia* spp., found across a wide geographical range (Europe, Asia and Africa). The cosmopolitan character of all these cestodes is attributed to the abundance and diversity of intermediate hosts and the lack of specificity for the definitive hosts [153]. Hence, the jackal, together with other carnivores, represents an important source of environmental contamination. Several of these species are known to be zoonotic, some with a minor impact (i.e. *D. caninum*), but other being a major public health threat (i.e. *E. granulosus*).

*Dipylidium caninum* occurs across the globe, human cases being reported in European and Asian countries after accidental ingestion of the infected cat and dog fleas with cysticercoid larvae [154]. Although the jackal is not a domestic species, hence not a direct source of infection to humans, it may transmit fleas to hunting, shepherd or stray dogs and participates together with other wild canids in the natural cycle of this cestode.

Several species of the genus *Mesocestoides* have been found in golden jackals in various regions. *Mesocestoides lineatus* is spread in Africa, Asia and Europe; it was rarely found in humans, with about 20 cases being described to date across the world [155]. Although the main definitive hosts are carnivores, humans can also act as accidental final hosts following ingestion of raw or undercooked meat of birds, amphibians, reptiles or small mammals [156]. The zoonotic potential of the other species of *Mesocestoides* in unknown.

The most well-represented family of tapeworms found in jackals is the Taeniidae. The high diversity of the Taeniidae in golden jackals reflects furthermore the wide range of mammalian prey species on which they feed. Golden jackals are hosts to both zoonotic species of *Echinococcus*. *Echinococcus granulosus* and *E*. *multilocularis* have been reported in this wild canid on multiple occasions and across a wide geographical range. The unilocular or cystic hydatidosis produced by larvae of *E*. *granulosus* (*sensu lato*) is a ubiquitous infection with high prevalence in various parts of the world [157]. Human multilocular or alveolar echinococcosis caused by *E*. *multilocularis* has recorded a significant increase in the incidence in northern Eurasia since 1990 [157, 158]. In several regions, high prevalences with both species were reported in golden jackals (Table 4). Reports of *Echinococcus* spp. in areas where this canid has recently spread or increased in abundance (i.e. central and eastern Europe) raise the important question on its role as a potentially new natural reservoir and infection source for humans and livestock.

Among species of genus *Taenia* and *Multiceps*, the most commonly reported species in golden jackals are *T. hydatigena*, *T. pisiformis*, *T. ovis* and *M. multiceps*. Other species (*T. polyacantha*, *T. taeniaeformis*, *T. krabbei*, *T. krepkogorski*, *T. crassiceps* and *M. serialis*) have been also found but only occasionally, mainly within the limited geographical range of Caucasus and central Asia (Table 4). The zoonotic potential of these species is limited, and only few human cases have been reported so far: *T*. *taeniaeformis* [159–162], *T*. *crassiceps* [163], *T. hydatigena* [164], *T. ovis* [165, 166], *M. multiceps* and *M*. *serialis* [167]. *Taenia krabbei*, *T*. *krepkogorski* and *T*. *polyacantha* are considered non-zoonotic tapeworms [158, 168]. Considering the common findings of a wide range of Taeniidae in golden jackals, the high spatial mobility of these hosts and the high resistance of taeniid eggs in the environment [169], the role of jackals as natural reservoirs and infection source for humans and domestic animals should be considered potentially important.

Species of *Spirometra* (Diphyllobothriidae) identified in golden jackals from Europe and Asia (*S*. *mansoni*, *S*. *houghtoni* and *S*. *erinaceieuropaei*) cause sparganosis in intermediate hosts. Humans may acquire the infection after drinking water contaminated with infected copepods or by ingestion of uncooked meat, and occasionally may lead to blindness, paralysis, and death [170, 171]. *Diphyllobothrium latum* is also reported in humans due to consumption of raw or undercooked fish, in cold water areas from the Holarctic Eurasia, overlaid to those regions where the species is recorded in jackal [172]. However, due to the limited number of reports, the role of golden jackals in the natural cycle of these diphyllobothriid cestodes remains unknown.

Tapeworm species with a limited geographic distribution are also reported in jackals. *Diplopylidium noelleri* and *Joyeuxiella* spp. are spread only in warm regions from Asia and Europe, probably due to the high abundance and diversity of reptiles, known as common intermediate hosts [173].

### Acanthocephalans

Although the diet of golden jackals generally includes invertebrates, wild birds, reptiles and small mammals which are intermediate or paratenic hosts in the life-cycle of thorny-headed worms [174–176], compared to other groups of helminths, there are only few and geographically limited reports of acanthocephalans in golden jackals. The diversity of acanthocephalans identified in this canid includes at least six species (Table 5) [176–181].

**Table 5:** Acanthocephalan parasites of the golden jackal, *Canis aureus*

Family	Species (synonym)	Origin	Prevalence (%)	Frequency	Intensity	Method	Reference
Class Archiacanthocephala							
Oligacanthorhynchidae	*Macracanthorhynchus* sp.	Iran	10.0	1/10		necropsy	[111]
	*Macracanthorhynchus catulinus*	Azerbaijan	0.8	1/114	24	necropsy	[137]
			17.0	14/82	1–6	necropsy	[99]
			na	na	na	necropsy	[100]
		Bulgaria	3.8	na	na	necropsy	[45]
		Kazakhstan	5.5	1/18	3	necropsy	[147]
		Russia	6.6	4/60	n/a	necropsy	[106]
			6.7	10/150	1–6	necropsy	[53]
		Tajikistan	na	na	na	necropsy	[148]
		Turkmenistan	na	na	na	necropsy	[177]
	*Macracanthorhynchus hirudinaceus* ^a^	Tunisia	na	1/5	na	necropsy	[149]
			3.2	1/31	6	necropsy	[140]
		Iran	case report		na	necropsy	[178]
			62.5	25/40	na	necropsy	[179]
			30.0	3/10	na	necropsy	[111]
			5.0	4/79	na	necropsy	[139]
			3.5	2/56	na	necropsy	[48]
	*Oncicola* sp.	Iran	na	na	na	necropsy	[178]
	*Oncicola canis*	Iran	case report		na	necropsy	[178]
			12.6	10/79	na	necropsy	[139]
	*Pachysentis canicola*	Iran	case report		na	necropsy	[180]
Class Palaeacanthocephala							
Centrorhynchidae	*Centrorhynchus itatsinis*	Azerbaijan	1.4	1/71	na	necropsy	[181]
			na	na	na	necropsy	[100]
*Incertae sedis*	*Echinorhynchus pachyacanthus*	Italy	na	na	na	necropsy	[138]

*Abbreviation*: *na* not applicable/not available/no answer

^a^Doubtful record

*Macracanthorhynchus catulinus* has been reported on several occasions in jackals in former USSR and Bulgaria, while the congeneric species *M. hirudinaceus* was found only in Tunisia and Iran. It is unclear if the reports of *M. hirudinaceus* (a parasite typically found in pigs; [155]) represent cases of pseudoparasitism or misidentifications with *M. catulinus* (a parasite typically found in canids), as most papers referring to these findings do not provide details on the identification methods. There are few scattered records of other carnivore-specific acanthocephalan species in golden jackals (*Oncicola canis*, *Pachysentis canicola*, *Centrorhynchus itatsinis* and *Echinorhynchus pachyacanthus*) in central Asia and Italy (Table 5).

### Nematodes

Nematodes constitute the most well-represented group of parasites in golden jackals, with 41 species identified (28 species in Chromadorea and 13 species in Enoplea) (Table 6) [182–256].

**Table 6:** Nematode parasites of the golden jackal, *Canis aureus*

Family	Species (synonym)	Origin	Prevalence (%)	Frequency	Intensity	Method	Reference
Phylum Nematoda							
Class Chromadorea							
Ancylostomatidae	*Ancylostoma* sp.	India^a^	na	3/3	na	CO	[182]
			na	5/5	na	CO	[183]
			31.6	19/60	na	CO	[144]
			case report		700 epg	CO	[184]
			case report		na	CO	[47]
		Iran^a^	na	2/2	10.5 epg	CO	[372]
	*Ancylostoma/Uncinaria* sp.	Bulgaria	84.6	na	na	necropsy	[141]
		India^a^	na	na	na	CO	[135]
		India	na	3/6	na	CO	[185]
		Iran	na	na	na	necropsy	[105]
		Serbia	33.3	20/60	na	necropsy	[49]
		Tunisia	na	5/5	na	necropsy	[149]
	*Ancylostoma braziliense* ^b^	India	na	na	na	na	[117]
	*Ancylostoma caninum*	Azerbaijan	17.3	17/98	1–20	necropsy	[99]
			na	na	na	necropsy	[100]
		Bangladesh	46.6	14/30	na	necropsy	[134]
		Bulgaria	54.5	6/11	na	necropsy	[142]
			11.5	na	na	necropsy	[45]
		Chechnya	50.0	8/16	3–18	necropsy	[103]
		Egypt	na	na	na	necropsy	[186]
		Greece	na	1/5	na	necropsy	[104]
		Hungary	40.0	8/20	na	necropsy	[59]
		India	na	na	na	na	[187]
			na	na	na	na	[143]
			na	na	na	na	[188]
		India^a^	na	na	na	CO	[189]
			100	12/12	100–400 epg	CO	[190]
		Iran	100	20/20	27.1	necropsy	[110]
			7.1	1/14	4	necropsy	[136]
			2.5	2/79	na	necropsy	[139]
			case report		na	necropsy	[155]
			3.5	2/56	na	necropsy	[48]
		Israel	33.3	5/15	na	necropsy	[146]
			76.0	13/17	50–800 epg	CO	[19]
		Russia	52.2	na	na	necropsy	[97]
			5.0	3/60	na	necropsy	[106]
			na	na	na	necropsy	[107]
			12.0	18/150	3–265	necropsy	[53]
		Serbia	0.2	1/447	2	necropsy	[108]
		Tajikistan	na	na	na	necropsy	[148]
		Tunisia	9.7	3/31	2–4	necropsy	[140]
		Uzbekistan	na	na	na	necropsy	[151]
	*Ancylostoma guentini*	India	na	na	na	necropsy	[191]
	*Placoconus lotoris* (syn. *Uncinaria lotoris*)^b^	Russia	16.6	2/12	4	necropsy	[98]
	*Uncinaria stenocephala*	Afghanistan	na	na	na	necropsy	[192]
		Azerbaijan	50.0	49/98	1–404	necropsy	[99]
			na	na	na	necropsy	[100]
		Bulgaria	64.0	na	na	necropsy	[101]
			na	na	na	necropsy	[237]
			45.4	5/11	na	necropsy	[142]
			84.6	na	na	necropsy	[45]
		Chechnya	50.0	8/16	4–23	necropsy	[103]
		Greece	na	4/5	na	necropsy	[104]
		Hungary	40.0	8/20	na	necropsy	[59]
		Iran	85.0	17/20	11.1	necropsy	[110]
			6.3	5/79	na	necropsy	[139]
		Russia	34.8	na	na	necropsy	[97]
			na	na	na	necropsy	[339]
			30.0	18/60	na	necropsy	[106]
			na	na	na	necropsy	[107]
			89.3	134/150	3–550	necropsy	[53]
		Tajikistan	na	na	na	necropsy	[148]
		Tunisia	68.0	21/31	1–54	necropsy	[140]
		Turkey	na	na	na	necropsy	[150, 194]
		Uzbekistan	na	na	na	necropsy	[151]
			na	na	na	necropsy	[152]
Ascarididae	*Baylisascaris devosi*	Azerbaijan	17.7	14/79	1–16	necropsy	[99]
	*Toxascaris* sp.	Kazakhstan	11.1	2/18	2–8	necropsy	[147]
	*Toxascaris leonina*	Afghanistan	na	na	na	necropsy	[192]
		Armenia^a^	case report		na	CO	[195]
		Azerbaijan	0.8	1/114	1	necropsy	[137]
			31.8	29/91	1–19	necropsy	[99]
			na	na	na	necropsy	[100]
		Bulgaria	36.0	na	na	necropsy	[101]
			5.8	na	na	necropsy	[141]
		Chechnya	100	16/16	1–12	necropsy	[103]
		Egypt	na	na	na	necropsy	[186]
		Hungary	15.0	3/20	na	necropsy	[59]
		Iran	30.0	3/10	na	necropsy	[111]
		Iran^a^	na	2/2	166.5 epg	CO	[372]
		Kazakhstan	11.1	2/18	2–11	necropsy	[147]
		Russia	43.5	na	na	necropsy	[97]
			10.0	2/20	4.00 ± 3.14	necropsy	[96]
			15.0	9/60	na	necropsy	[106]
			na	na	na	necropsy	[98]
			na	na	na	necropsy	[107]
			4.7	7/150	2–23	necropsy	[53]
		Tajikistan	na	na	na	necropsy	[148]
		Tunisia	6.5	2/31	1–7	necropsy	[140]
		Turkey	na	na	na	necropsy	[150]
		Uzbekistan	na	na	na	necropsy	[151]
			na	na	na	necropsy	[152]
	*Toxocara* sp.	India^a^	21.6	13/60	na	CO	[144]
			na	na	na	CO	[196]
			na	2/2	40–700 epg	CO	[197]
			na	3/6	na	CO	[185]
	*Toxocara canis* (syn. *Belascaris marginata*)	Azerbaijan	32.6	32/98	1–21	necropsy	[99]
			na	na	na	necropsy	[100]
		Bangladesh	40.0	12/30	na	necropsy	[134]
			na	2/5	na	CO	[118]
		Bulgaria	54.5	6/11	na	necropsy	[142]
			7.7	na	na	necropsy	[45]
		Chechnya	50.0	8/16	3–18	necropsy	[103]
		Greece	na	2/5	na	necropsy	[104]
		Hungary	20.0	4/20	na	necropsy	[59]
		India (as *B. marginata*)	na	na	na	na	[187]
			na	na	na	na	[143]
			na	na	na	na	[112]
		India^a^	na	na	na	CO	[189]
		Iran	10.0	2/20	na	necropsy	[110]
			7.1	1/14	10	necropsy	[136]
			10.0	1/10	na	necropsy	[111]
			5.0	4/79	na	necropsy	[139]
			12.5	7/56	na	necropsy	[48]
		Iran^a^	na	2/2	25 ± 5 epg	CO	[372]
		Russia	60.9	na	na	necropsy	[97]
			5.0	1/20	2.00 ± 2.13	necropsy	[96]
			6.6	4/60	na	necropsy	[106]
			na	na	na	necropsy	[98]
			na	na	na	necropsy	[107]
			23.3	35/150	1–22	necropsy	[53]
		Serbia	1.6	7/447	7.8 ± 2.1	necropsy	[108]
			23.3	14/60	na	necropsy	[49]
		Tajikistan	na	na	na	necropsy	[148]
		Tunisia	16.0	5/31	1–9	necropsy	[140]
		Turkey	na	na	na	necropsy	[150]
		Uzbekistan	na	na	na	necropsy	[151]
			na	na	na	necropsy	[152]
	*Toxocara cati* (syns *Ascaris mystax*, *T. mystax*)^b^	Italy	na	na	na	necropsy	[138]
		Russia	26.1	na	na	necropsy	[97]
			5.0	1/20	2.0 ± 1.9	necropsy	[96]
		Uzbekistan	na	na	na	necropsy	[152]
Crenosomatidae	*Crenosoma vulpis*	Azerbaijan	14.6	12/82	1–19	necropsy	[99]
			na	na	na	necropsy	[100]
		Bangladesh	na	1/5	na	necropsy	[118]
		Chechnya	100	16/16	10–23	necropsy	[103]
		Hungary	30.0	6/20	na	necropsy	[59]
		Russia	26.1	n/a	na	necropsy	[97]
			10.0	6/60	na	necropsy	[106]
			na	na	na	necropsy	[107]
			12.0	18/150	6–297	necropsy	[53]
	*Crenosoma petrowi*	Chechnya	37.5	6/16	16–38	necropsy	[103]
Diaphanocephalidae	*Kalicephalus schadi fotedari* ^b^	India	na	na	na	necropsy	[198]
Dracunculidae	*Dracunculus medinensis*	India	na	na	na	na	[112]
		Kazakhstan	16.6	3/18	2–4	necropsy	[147]
		Tajikistan	na	na	na	necropsy	[148]
Gnathostomatidae	*Gnathostoma spinigerum*	India, Bangladesh, Burma	na	na	na	na	[112]
		Bangladesh	26.6	8/30	na	necropsy	[134]
Gongylonematidae	*Gongylonema* sp.^b^	Serbia	0.4	2/447	1	necropsy	[108]
Metastrongylidae	*Angiostrongylus vasorum*	na	na	1/1	na	EI	[199]
		Hungary	10.0	2/20	na	necropsy	[59]
Molineidae	*Molineus patens*	Russia	8.3	5/60	na	necropsy	[106]
			26.0	39/150	3–104	necropsy	[53]
Onchocercidae	*Acanthocheilonema reconditum* (syn. *Dipetalonema reconditum*)	Bulgaria	30.0	3/10	na	K	[200]
		Kenya (as *Dipetalonema reconditum*)	na	na	na	na	[201]
	*Dirofilaria immitis*	Azerbaijan	na	na	na	necropsy	[100]
		Bangladesh	na	4/5	na	necropsy	[118]
		Bulgaria	8.9	5/56	2–16	necropsy/K	[202]
			9.6	na	na	necropsy	[45]
			70.0	7/10	na	K	[200]
			37.5	122/325	1–19	necropsy	[203]
		Greece	case report		4	necropsy	[204]
		Hungary	7.4	2/27	1–10	necropsy	[205]
		India^a^	na	na	na	na	[206]
		Iran	28.5	4/14	13	necropsy	[136]
			na	na	na	necropsy	[207]
			11.4	9/79	na	necropsy	[139]
			1.7	1/56	na	necropsy	[48]
			8.9	4/45	1–10	necropsy, molecular	[208]
		Romania	18.52	10/54	1–7	necropsy	[209]
			9.26	5/54	na	molecular	[209]
		Russia	8.3	1/12	29	necropsy	[98]
			8.3	5/60	na	necropsy	[106]
			20.0	na	na	necropsy	[339]
			22.7	34/150	2–23	necropsy	[53]
		Serbia	7.3	32/437	na	necropsy	[210]
		Uzbekistan	na	na	na	necropsy	[152]
	*Dirofilaria repens*	Egypt	na	na	na	necropsy	[186]
		Iran	10.0	2/20	na	necropsy	[110]
		Romania	1.8	1/54	na	molecular	[209]
		Russia	3.3	2/60	na	necropsy	[106]
		Uzbekistan	na	na	na	necropsy	[152]
Oxyuridae	*Syphacia* sp.^b^	Kazakhstan	5.5	1/18	7	necropsy	[147]
Physalopteridae	*Physaloptera* sp.	Iran	na	na	na	necropsy	[105]
		Kazakhstan	5.5	1/18	4	necropsy	[147]
	*Physaloptera sibirica*	Azerbaijan	na	na	na	necropsy	[100]
		Kazakhstan	11.1	2/18	1–3	necropsy	[147]
Rictulariidae	*Rictularia* sp.	Egypt	na	na	na	necropsy	[186]
		Greece	na	1/5	na	necropsy	[104]
	*Rictularia affinis* (syn. *Pterygodermatites affinis*)	Azerbaijan	36.3	28/77	1–29	necropsy	[99]
			na	na	na	necropsy	[100]
		Bulgaria	7.7	na	na	necropsy	[45]
		India (as *P. affinis*)	na	na	na	na	[117]
			na	na	na	na	[188]
			na	na	na	na	[112]
		Iran	50.0	5/10	na	necropsy	[111]
			5.0	4/79	na	necropsy	[139]
		Kazakhstan	11.1	2/18	1–3	necropsy	[147]
		Tunisia (as *P. affinis*)	6.5	2/31	1	necropsy	[140]
		Turkmenistan	na	na	na	necropsy	[177]
		Uzbekistan	na	na	na	necropsy	[151]
			na	na	na	necropsy	[152]
	*Rictularia cahirensis*	Egypt	na	na	na	necropsy	[186]
		Iran	10.0	2/20	na	necropsy	[110]
		Turkey	na	na	na	necropsy	[340]
		Uzbekistan	na	na	na	necropsy	[151]
Spirocercidae	*Spirocerca arctica*	Azerbaijan	16.6	2/12	5–14	necropsy	[99]
			na	na	na	necropsy	[100]
	*Spirocerca lupi* (syn. *Filaria sanguinolenta*)	Azerbaijan	23.4	23/98	1–29	necropsy	[99]
			na	na	na	necropsy	[100]
		India	case report		na	necropsy	[211]
			na	na	na	na	[112]
		India^a^	case report		na	necropsy	[212]
		Iran	2.5	2/79	na	necropsy	[139]
		Italy (as *F. sanguinolenta*)	na	na	na	necropsy	[138]
		Uzbekistan	na	na	na	necropsy	[151]
	*Spirocerca sanguinolenta*	India	na	na	na	necropsy	[213]
Strongyloididae	*Strongyloides* sp.	India^a^	na	na	na	CO	[214]
		Iran^a^	na	2/2	5.5 ± 0.7 epg	CO	[372]
	*Strongyloides stercoralis*	Iran	na	na	na	necropsy	[105]
Subuluridae	*Oxynema linstowi*	Tunisia	3.2	1/31	2	necropsy	[140]
Thelaziidae	*Thelazia callipaeda*	Romania	1.6	1/64	70	necropsy	[215]
Class Enoplea							
Capillariidae	*Capillaria* sp.	India^a^	na	na	na	CO	[214]
		Russia	2.7	4/150	1–45	necropsy	[53]
	*Eucoleus aerophilus* (syn. *Thominx aerophilus*)	Azerbaijan (as *T. aerophilus*)	na	na	na	necropsy	[100]
		Hungary	5.0	1/20	na	necropsy	[59]
		Russia	8.3	5/60	na	necropsy	[106]
			37.3	56/150	1–6	necropsy	[53]
	*Capillaria boehmi*	Russia	30.0	45/150	1–11	necropsy	[53]
	*Capillaria plica*	Azerbaijan	14.8	4/27	1–4	necropsy	[99]
			na	na	na	necropsy	[100]
		Bulgaria	16.4	na	na	necropsy	[45]
		Hungary	45.0	9/20	na	necropsy	[59]
		Russia	11.6	7/60	na	necropsy	[106]
			33.3	50/150	1–123	necropsy	[53]
	*Capillaria putorii*	Azerbaijan	na	na	na	necropsy	[100]
Dioctophymatidae	*Dioctophyme renale*	Azerbaijan	3.5	4/114	1	necropsy	[137]
		Iran	35.0	7/20	na	necropsy	[110, 216]
		Russia	3.3	2/60	na	necropsy	[106]
		Tajikistan	na	na	na	necropsy	[148]
		Uzbekistan	na	na	na	necropsy	[151]
			na	na	na	necropsy	[152]
	*Dioctophyme skrjabini*	Russia	17.4	na	na	necropsy	[97]
Trichinellidae	*Trichinella* sp.	Armenia	33.3	5/15	na	TRIC	[217]
		Azerbaijan	30.1	na	na	TRIC	[218]
			28.6	na	na	TRIC	[219]
		Bulgaria	50.0	na	na	TRIC	[220]
			33.3	15/45	na	TRIC	[221]
			21.2	7/33	na	TRIC	[222]
		Croatia	na	na	na	AD	[102]
		Georgia	80.0	na	na	TRIC	[218]
		Romania	53.7	29/54	na	TRIC	[223]
		Russia	14.3	na	na	TRIC	[224]
			case report		na	AD	[225]
			25.0	75/302	na	TRIC	[226]
			case report		na	AD	[227]
		Tajikistan	na	na	na	TRIC	[228]
		Thailand	case report		na	TRIC	[229]
			case report		na	TRIC	[230]
		USSR (former)	36.5	na	na	TRIC	[231]
		Yugoslavia (former)	25.0	na	na	TRIC	[232]
	*Trichinella britovi*	Algeria	case report		na	PCR	[233]
		Azerbaijan	case report		na	AD	[234]
		Iran	11.1	2/18	na	AD, PCR	[235]
		Kazakhstan	na	na	na	TRIC	[234]
		Lithuania	na	3/4	0.9–1.4 lpg	AD, PCR	[236]
		Romania	case report		55 lpg	AD, PCR	[237]
		Serbia	4.7	2/42	na	AD, PCR	[238]
			15.4	2/13	na	AD, PCR	[239]
			27.8	25/90	1.1–57.6 lpg	AD, PCR	[240]
		Turkmenistan	case report		na	AD	[234]
		Uzbekistan	case report		na	AD	[234]
	*Trichinella nativa*	Kazakhstan	18.9	4/18	7 lpg	AD	[147]
		Lithuania	n/a	1/4	0.9–1.4 lpg	AD, PCR	[236]
		URSS (former)	61.5	na	na	TRIC	[241]
	*Trichinella nelsoni*	Iran	na	2/2	na	TRIC	[242]
		Kazakhstan	16.2	3/18	6 lpg	na	[147]
	*Trichinella pseudospiralis*	Russia	2.7	4/150	na	TRIC	[53]
	*Trichinella spiralis*	Afghanistan	case report		214 lpg	TRIC	[243]
		Azerbaijan	20.4	17/83	1–7 lpg	TRIC	[244]
			21.1	25/114	1–22 lpg	TRIC	[137]
			17.5	16/91	1–44 lpg	TRIC	[99]
		Bulgaria	na	na	na	TRIC	[245]
			45.0	na	na	TRIC	[101]
			40.0	na	na	AD	[45]
		Georgia	36.6	na	na	TRIC	[246]
		Hungary	9.0	1/11	na	AD, PCR	[365]
		Iran	60.3	38/63	na	TRIC	[247]
			55.5	10/18	na	TRIC	[248]
			na	3/3	na	TRIC	[249]
			55.7	59/106	na	TRIC	[250]
			84.0	na	na	TRIC	[251]
		Kazakhstan	5.5	1/18	3 lpg	TRIC	[147]
		Russia	43.5	na	na	TRIC	[97]
			21.6	13/60	na	TRIC	[106]
			55.3	83/150	na	TRIC, AD	[53]
		Senegal	33.0	na	na	TRIC	[252]
		Serbia	na	3/3	1.9–21.4 lpg	AD, PCR	[253]
			8.3	1/12	3 lpg	AD, PCR	[254]
			7.9	3/38	3 lpg	AD, PCR	[255]
			14.2	6/42	na	AD, PCR	[238]
			38.4	5/13	na	AD, PCR	[239]
			71.1	64/90	0.59–152.8 lpg	AD, PCR	[240]
		Tunisia	na	2/2	na	TRIC	[256]
Trichuroididae	*Trichuris* sp.	India^a^	15.0	9/60	na	CO	[144]
	*Trichuris georgicus* (syn. *Trichocephalus georgicus*)	Azerbaijan	14.4	12/83	1–12	necropsy	[99]
			na	na	na	necropsy	[100]
	*Trichuris vulpis* (syn. *Trichocephalus vulpis*)	Azerbaijan (as *Trichocephalus vulpis*)	11.2	11/98	1–11	necropsy	[99]
			na	na	na	necropsy	[100]
		Bangladesh	na	1/5	na	CO	[118]
		Bulgaria	30.7	na	1–156	necropsy	[141]
			36.3	4/11	na	necropsy	[142]
		Russia (as *Trichocephalus vulpis*)	21.6	13/60	na	necropsy	[106]
			35.3	53/150	1–70	necropsy	[53]
		Serbia	11.6	4/60	na	necropsy	[49]
		Hungary	10.0	2/20	na	necropsy	[59]

*Abbreviations: AD* artificial digestion, *CO* coprological examination, *EI* experimental infection, *epg* eggs per gram faeces, *lpg* larvae per gram tissue, *K* Knott test, *PCR* polymerase chain reaction, *TRIC* trichinelloscopy, *na* not applicable/not available

^a^Animals kept in captivity

^b^Doubtful record

#### Ascarids

Ascarids, primarily considered heteroxenous nematodes, have lost their intermediate hosts and have adapted to direct transmission or through paratenic hosts [257]. Four species are reported in golden jackals, with *Toxocara canis* and *Toxascaris leonina* being ubiquitous. *Baylisascaris devosi* is a species typically found in mustelids inhabiting the northern hemisphere [258]. Its presence in golden jackals has been reported only once, in Azerbaijan [99]. This broad distribution and common presence of ascarids in this wild canid can be explained by the intervention of numerous paratenic hosts, possible preys for jackals, in the life-cycle of these nematode species (mostly rodents and invertebrates such as earthworms and insects) [259].

#### *Strongyloides*

The cosmopolitan and zoonotic *Strongyloides stercoralis* infects about 200 million people, more commonly in tropical and subtropical climates [260]. Despite the large number of records in domestic dogs from various countries, the species has been reported only once in golden jackals (Table 6). The lack of reports in other parts of the jackal’s range could be explained by a low receptivity of this host or by failures of finding the parasites during necropsy due to their small size. A moderate prevalence of 5.6% is also recorded in dogs from northeastern Iran [261] which is higher than estimated prevalence in humans across the country that ranges between 0.1 and 0.3% [262]. Although carnivores can be a source of infection for humans via larvae that develop in the environment, the principal reservoirs of *S*. *stercoralis* are humans. The role of domestic and wild carnivores in the epidemiology of strongyloidiasis remains to be clarified [155].

#### Hookworms and other digestive tract strongylids

Several hookworm (Ancylostomatidae) species have been reported in golden jackals, with *Ancylostoma caninum* and *Uncinaria stenocephala* commonly reported across the entire geographical range of these hosts. Additionally, *A*. *guentini* was described from golden jackals in India, being so far the only known host for this parasite [191]. The remaining two records (*Placoconus lotoris*, known otherwise only from new world procyonids, and *Ancylostoma braziliense*, typically found in the Americas) we list as doubtful and as these are probably misidentifications of other hookworm species. The opportunistic behaviour of the golden jackals leads them to venture close to human habitats to feed [2]. The proximity with domestic dogs allows interspecific transmission of ancylostomid species, due to the high rate of soil and grass contamination [263]. In this regard, increased prevalence recorded in Russia, ranging between 5.0 and 52.2%, is correlated with similar values in dogs, between 2.06 and 62.3% [264]. *Ancylostoma caninum* and *U. stenocephala* possess a zoonotic potential causing dermal larva migrans in humans [260]. Although a direct relationship between the numerous reports in golden jackals and the presence of disease in humans cannot be established, this carnivore species probably contributes to the presence of Ancylostomatidae larvae in rural and periurban areas.

*Molineus patens* is a hookworm commonly reported in a wide range of carnivores in the Palaearctic and Nearctic [265], including two records from jackals in Russia. However, its zoonotic role has not been documented.

#### Metastrongyloids

Compared to foxes, little is known about the respiratory and cardiovascular strongylids of golden jackals. *Crenosoma vulpis* has been reported on several occasions in Asia and Europe, with variable prevalence (Table 6). There is a single report of *Crenosoma petrowi* in golden jackals, a parasite otherwise known mainly from mustelids in North America [266], one report from bears (also in North America) and another one from stone martens in Italy [267]. Surprisingly, there is only one record of *Angiostrongylus vasorum* in golden jackals, suggesting either a lack of habitat overlap or a low detection sensitivity during necropsy.

#### Filarioids

Zoonotic filarioids *Acanthocheilonema reconditum*, *Dirofilaria immitis* and *D*. *repens* have been all reported in golden jackals in various countries (Table 6). They have been found both as adults during necropsies but also as microfilariae demonstrating the reservoir role of jackals. *Dirofilaria* spp. are responsible in humans of conjunctivitis, focal pulmonary infarction with granuloma formation and subcutaneous and submucosal lesions in the lung and conjunctiva [268–270]. A recent review listed 1782 human *Dirofilaria* spp. infections, out of which 372 were pulmonary (in Australia, North and South America) and 1410 were subcutaneous/ocular cases (mostly in Europe and Asia) [271]. *Acanthocheilonema reconditum* is considered non-pathogenic for canids [155], but a single human case is well documented as being caused by this species and at least other two, by other *Acanthocheilonema* species [270, 272, 273]. The recorded prevalence of *D*. *immitis* has significantly varied in different areas between 7.3% in Serbia and 80.0% in Bangladesh, and seems to be consistent with the prevalence registered in dogs, generally between 40 and 70% in endemic areas [155]. The prevalence of *D*. *repens* in golden jackals ranged between 3.3 and 10.0%, resembling that recorded in dogs, generally varying from 5 to 20% [155].

The oriental eye-worm *Thelazia callipaeda* has been identified in golden jackals only in Romania [215], but this is probably due to lack of proper examinations of the eyes during the necropsy in other studies rather than a resistance of this host. Dogs originating in the Far East were initially considered the main host of the nematode [257]. Over the last 15 years, *T*. *callipaeda* has shown an increase in the distribution area mainly in Europe, with many new host records [274]. Human thelaziosis followed the same geographical spreading, with recent cases of infection diagnosed [274]. In this epidemiological picture, the golden jackal occurs as a new reservoir host.

#### Capillariids

Respiratory capillariids of carnivores (*C*. *aerophila*, *C*. *boehmi* and *C*. *putorii*) are considered primarily homoxenous, but the earthworms often act as facultative intermediate hosts [1, 275]. Along with the heteroxenous species *Capillaria plica* found in the urinary bladder, all species are cosmopolitan [257]. They have been found in golden jackals in Russia and other former Soviet Union countries. Only *C*. *plica* and *C*. *aerophila* are known to be zoonotic [276, 277], but their public health impact is minor. As the primary source of infection with *Capillaria* is the soil contaminated by infective eggs [155], the jackal can play an epidemiological role and secondary source of infection for domestic carnivores and humans.

#### *Trichinella*

Trichinellids are an important group of meat-borne zoonotic parasites [278] for which the golden jackals represent an important reservoir. Five species, *T*. *britovi*, *T*. *nativa*, *T*. *nelsoni*, *T*. *pseudospiralis* and *T*. *spiralis*, were recorded in mountainous and lowland regions across the distribution range of the golden jackal in Europe and Asia. However, only three of these (*T. britovi*, *T. nativa* and *T. spiralis*) have been confirmed molecularly (Table 6). We consider all the records by artificial digestion or trichinelloscopy (see Table 6) where the species is named as hypothetic, as there is no reliable morphological means of differentiation between species, hence we recommend to consider these as *Trichinella* sp. Nevertheless, the zoonotic potential have been shown for most *Trichinella* species, hence, the golden jackal represents an important natural sylvatic reservoir for these nematodes [240].

#### Other nematodes

Various other groups of nematodes have been found in golden jackals (families Spirocercidae, Dracunculidae, Gnathostomatidae, Physalopteridae, Rictulariidae, Subuluridae), but the reports are occasional (Table 6). Other groups (*Kalicephalus*, *Syphacia* and *Gongylonema*) are with high probability pseudoparasites, originating in prey hosts.

*Spirocerca lupi* is a rare zoonotic nematode species also identified in golden jackals in Europe, central and southern Asia (Table 6). Although a single human infection has been reported [279], the jackal may represent a reservoir host that maintains the life-cycle of the parasite in a certain region. Two other species of the genus *Spirocerca* (*S. arctica* and *S. sanguinolenta*, both described from domestic dogs) have been also reported in jackals, but their taxonomic status and biology are unknown.

*Dracunculus medinensis* has been identified in the golden jackal from several central and southern Asian countries. Currently the disease in humans has been declared extinct in the vast majority of the countries, with only three (Chad, South Sudan and Ethiopia) reporting cases in 2016 [280]. Dogs are considered to be important reservoirs for human infection [155, 281, 282]. In 2016, more than 1000 dogs in Chad, 14 dogs in Ethiopia, and 11 dogs in Mali were reported with guinea-worm [280]. In this context, understanding the role of wild canids (including golden jackals) remains a crucial aspect in the management of the ongoing eradication campaign.

Another zoonotic species identified in golden jackals from tropical Asian countries (India, Bangladesh, Myanmar) is *Gnathostoma spinigerum*. Gnathostomiasis, a major food-borne parasitic zoonosis and a significant public health problem, is considered an emerging imported disease in Europe and a common human infection in central and South America, and Asia [283]. Domestic and wild mammals are the final hosts and numerous intermediate and paratenic hosts are the source for the human infection. The golden jackals maintain the sylvatic focus of the parasites and interfere with the domestic cycle, at least in several Asian countries (Table 6).

Various other carnivore-specific spirurids have been found in golden jackals (*Physaloptera sibirica*, *Rictularia affinis*, *R. cahirensis* and *Oxynema linstowi*), but the role of this host species in their natural cycle remains unknown.

The cosmopolitan species *Dioctophyme renale* causes a severe kidney destruction in the carnivore definitive hosts. Although with limited zoonotic importance, so far around 20 human cases have been reported [284]. American minks seem to be the main reservoirs of the parasite [257], but an increased prevalence is also recorded in other wild and domestic carnivores. In golden jackals, *D. renale* has been reported in Asia, where the prevalence ranged between 3.3–35.0% (Table 6). Interestingly, this wild canid has shown a twice higher prevalence than stray dogs in the same geographical region [110], demonstrating the role of the jackal in the development of parasite’s cycle in nature.

*Trichuris vulpis* has been found on various occasions in golden jackals in Europe and Asia (Table 6). The high prevalence of *T*. *vulpis* infection in golden jackals (10.0–36.3%) is in line with the value recorded in dogs originating from the same areas: 25% in India [285], 20% in Bulgaria [286] and 8.95% in Russia [264]. Although the prevalence of *T*. *vulpis* in domestic and wild canids is generally high, only around 60 human cases have been recorded [155].

## Arthropods

A great variety of Arthropods have been found in golden jackals (Table 7) [287–328].

**Table 7:** Arthropod parasites of the golden jackal, *Canis aureus*

Family	Species	Origin	Prevalence (%)	Frequency	Intensity (A, ♂, ♀, N, L)^b^	Reference
Class Arachnida						
Demodecidae	*Demodex* spp*.*	Israel	na	1/1	na	[287]
	*Demodex canis*	Russia	3.3	5/150	na	[53]
	*Demodex folliculorum*	Bangladesh	na	na	na	[118]
Ixodidae	*Amblyomma* sp.	Nepal	na	na	na	[288]
	*Amblyomma varanense* (syn. *Aponomma gervaisi lucasi*)	India	na	na	na	[289]
	*Amblyomma variegatum*	Haute-Volta	na	na	7 N	[290]
		Senegal	na	na	54 L	[291]
	*Dermacentor marginatus*	Russia	15.3	23/150	1–26	[53]
		Serbia	45.0	9/20	na	[292]
	*Dermacentor reticulatus*	Austria	na	1/1	17 ♂; 2 ♀	[56]
		Hungary	na	4/4	10 A	[293]
		Italy	na	1/1	na	[116]
		Romania	12.6	10/79	46 ♂; 25 ♀	[294]
		Russia	62.0	93/150	1–26	[53]
	*Haemaphysalis* sp*.*	Iran	1.7	1/56	na	[48, 295, 296]
		Nepal	na	na	na	[288]
	*Haemaphysalis adleri*	Iraq	na	na	na	[297]
		Israel	na	2/2	4 ♂; 1 ♀	[298]
			na	3/3	na	[299]
	*Haemaphysalis bispinosa*	India	na	na	na	[300]
		Nepal	na	na	na	[288]
	*Haemaphysalis canestrinii*	India	na	1/1	9 ♂; 3 ♀,	[301]
			na	na	na	[300]
		Pakistan	na	3/3	3 ♂; 1 ♀	[301]
	*Haemaphysalis concinna*	Austria	na	1/1	1 N	[56]
		Hungary	na	4/4	7 N; 4 L	[293]
		Romania	1.2	1/79	1 N	[294]
	*Haemaphysalis flava*	India	na	na	A	[289]
	*Haemaphysalis indoflava*	India	na	na	na	[302]
	*Haemaphysalis intermedia*	India	na	2/2	8 ♂; 17♀; 28 N; 13 L	[303]
			na	na	na	[300]
	*Haemaphysalis kutchensis*	India	na	na	10 ♂; 1 ♀	[348]
	*Haemaphysalis leachii* (syns *H. leachii leachii*, *H. leachii indica*)	Egypt (as *H. leachii leachii*)	na	na	na	[305]
		India	na	1/1	9 ♂; 3 ♀	[306]
		India (as *H. leachii indica*)	na	na	na	[289]
			na	2/2	7 ♂; 2♀; 4 N; 1 L	[307]
		Nepal (as *H. leachii indica*)	na	3/3	6 N	[307]
	*Haemaphysalis longicornis* (syn. *H. neumanni*)	Ceylon	na	na	na	[308]
	*Haemaphysalis paraleachi*	Sudan	100	10/10	42 ♂; 4 ♀	[309]
	*Haemaphysalis parva* (syn. *H. otophila*)	India	na	na	na	[310]
		Israel	na	3/3	na	[299]
		Israel (as *H. otophila*)	na	6/6	37	[311]
	*Haemaphysalis punctata*	Romania	na	4/4	na	[312]
			2.5	2/79	1 ♂; 2 N	[294]
	*Hyalomma* sp.	Russia	0.7	1/150	1	[53]
		Tajikistan	na	na	na	[148]
	*Hyalomma aegyptium*	USSR (former)	na	na	na	[313]
	*Hyalomma anatolicum*	Tajikistan	na	na	na	[148]
	*Hyalomma asiaticum*	Tajikistan	na	na	na	[148]
	*Hyalomma scupense*	Tajikistan	na	na	na	[148]
	*Ixodes* sp.	Iran	na	1/1	na	[314]
		Russia	na	na	na	[339]
		Tajikistan	na	na	na	[148]
	*Ixodes acuminatus* (syn. *I. redikorzevi theodori*)	Israel	na	6/6	1	[311]
	*Ixodes canisuga*	Hungary	na	4/4	1 N	[293]
	*Ixodes hexagonus*	Romania	10.1	8/79	2 ♂; 11 ♀; 24 N; 12 L	[294]
	*Ixodes ovatus*	Nepal	na	1/1	6 ♀	[315]
	*Ixodes ricinus*	Hungary	na	4/4	3 A	[293]
		Iran	3.5	2/56	na	[48, 295, 296]
		Italy	na	1/1	na	[116]
		Romania	na	1/1	na	[316]
			na	4/4	na	[312]
			26.5	21/79	54 ♂; 45♀; 3 N; 4 L	[294]
		Russia	68.7	103/150	1–62	[53]
		Serbia	55.0	11/20	na	[292]
	*Rhipicephalus* sp.	Iran	1.7	1/56	na	[48, 295, 296]
		Tajikistan	na	na	na	[148]
	*Rhipicephalus annulatus* (syn. *Boophilus calcaratus*)	Russia	1.3	2/150	1–2	[53]
	*Rhipicephalus cuspidatus*	Haute-Volta	na	na	na	[290]
	*Rhipicephalus haemaphysaloides*	India	na	na	na	[289]
			na	1/1	4 ♂	[306]
			na	2/2	1 ♀; 2 N	[303]
			na	na	na	[300]
		Nepal	na	na	na	[288]
	*Rhipicephalus leporis*	Iraq	na	na	na	[297]
		Tajikistan	na	na	na	[148]
	*Rhipicephalus pumilio*	Tajikistan	na	na	na	[148]
		Uzbekistan	na	na	na	[152]
	*Rhipicephalus rossicus*	Tajikistan	na	na	na	[148]
	*Rhipicephalus sanguineus*	Algeria	na	2/2	15 ♂; 8 ♀	[317]
		Burma, Ceylon, India	na	na	na	[289]
		India	na	na	na	[300]
		Iran	na	na	na	[314]
		Israel	na	6/6	na	[311]
		Nepal	na	na	na	[288]
		Nigeria^a^	na	3/6	na	[41]
		Romania	na	1/1	na	[316]
			na	4/4	na	[312]
			1.2	1/79	1 ♂	[294]
		Serbia	0.5	1/20	na	[292]
		Tajikistan	na	na	na	[148]
		Turkey	na	na	na	[150]
			na	na	na	[318]
	*Rhipicephalus schulzei*	Tajikistan	na	na	na	[148]
	*Rhipicephalus simus*	Kenya	na	na	na	[319]
	*Rhipicephalus sulcatus*	Haute-Volta	na	na	5 ♂	[290]
	*Rhipicephalus turanicus*	Iraq	na	na	na	[320]
			100	14/14	na	[297]
		Tajikistan	na	na	na	[148]
		Uzbekistan	na	na	na	[152]
Psoroptidae	*Otodectes cynotis*	Iran	1.7	1/56	na	[48, 295, 296]
Sarcoptidae	*Sarcoptes scabiei*	Israel	na	1/1	na	[287]
Class Insecta						
Boopiidae	*Heterodoxus spiniger*	Africa (North, East); Asia (South); Europe (Southeast)	na	na	na	[321]
		Uganda	na	1/1	1 ♂; 2 ♀	[322]
Ceratophyllidae	*Paraceras melis*	Russia	10.0	15/150	1–12	[53]
			3.3	5/150	na	[323]
Coptopsyllidae	*Coptopsylla lamellifer dubinini*	Uzbekistan	na	na	na	[152]
Hippoboscidae	*Hippobosca longipennis*	Russia	2.7	4/150	1–2	[53]
Linognathidae	*Linognathus setosus*	Afrotropical Region	na	na	na	[324]
		Nepal	na	na	na	[288]
Pediculidae	*Pediculus* sp.	Bangladesh	na	na	na	[118]
Pulicidae	*Pulex irritans*	Afghanistan	na	na	2 ♂; 1 ♀	[325]
		Iran	na	na	3 ♂; 1 ♀	[326]
		Israel	na	6/6	36	[311]
		Russia	24.0	36/150	1–15	[53]
			14.0	21/150	na	[323]
		Tajikistan	na	na	na	[148]
		Uzbekistan	na	na	na	[152]
	*Ctenocephalides canis*	Afghanistan	na	na	10 ♂; 38 ♀	[325]
		Iran	na	na	13 ♂; 25 ♀	[326]
			10.8	6/56	na	[48, 295, 296]
		Israel	na	4/6	27	[311]
		Nigeria^a^	na	1/6	na	[41]
		Russia	48.0	72/150	1–28	[53]
			17.3	30/150	na	[323]
		Tajikistan	na	na	na	[148]
		Turkey	na	na	na	[150]
		Uzbekistan	na	na	na	[152]
	*Ctenocephalides felis* (syn. *C. felis felis*)	Ethiopia	na	na	na	[327]
		India (as *C. felis felis*)	na	na	na	[328]
		Iran (as *C. felis felis*)	na	na	2 ♂; 2 ♀	[326]
		Israel	50.0	3/6	6	[311]
		Russia	3.3	5/150	1–4	[53]
		Tajikistan	na	na	na	[148]
		Uzbekistan	na	na	na	[152]
	*Echidnophaga gallinacea*	Afghanistan	na	na	1 ♂	[325]
		Ethiopia	na	na	na	[327]
	*Xenopsylla nesokiae*	Tajikistan	na	na	na	[148]
Trichodectidae	*Trichodectes canis*	Russia	13.3	20/150	1–34	[53]
		Tajikistan	na	na	na	[148]
Vermipsyllidae	*Chaetopsylla globiceps*	Russia	27.3	41/150	1–19	[53]
			6.7	10/150	na	[323]
Class Maxillopoda						
Linguatulidae	*Linguatula serrata*	Romania	1.3	1/73	1 ♂	Our unpublished data

*Abbreviation: na* not applicable/not available

^a^Animals kept in captivity

^b^A, adults; ♂, male; ♀, female; N, nymphs; L, larvae

### Ticks

Due to the large geographical range, the diversity of ticks parasitizing golden jackals is high. Ticks from 37 species belonging to six genera have been recorded in jackals throughout Europe, Asia and Africa. Nevertheless, the number of studies on tick-borne pathogens is surprisingly low. The common tick species found in golden jackals in Europe, i.e. *Dermacentor reticulatus*, *D. marginatus*, *Haemaphysalis concinna*, *H. punctata*, *Ixodes canisuga*, *I. hexagonus*, *I. ricinus* and *Rhipicephalus sanguineus* (*s.l*.), show that they share these ticks with other wild canids, like foxes [329] or with domestic dogs [330]. The two most commonly reported ticks in golden jackals from Europe are *D. reticulatus* and *I. ricinus*. These ticks are known to be important vectors for *Babesia canis* and important tick-borne bacteria, *Borrelia burgdorferi* (*s.l*.) and *Anaplasma phagocytophilum*. However, the reports of these pathogens in *C. aureus* are scarce (a single report of *Babesia canis* from Romania [42]). In Asia, the most common ticks on jackals are several species of genus *Haemaphysalis*, with a high diversity of species reported: *H. leachi*, *H. adleri*, *H. bispinosa*, *H. canestrinii*, *H. flava*, *H. indoflava*, *H. intermedia*, *H. kutchensis* and *H. parva*. However, studies on the pathogens they might transmit are absent. Several of these *Haemaphysalis* species are shared with domestic dogs or other wild carnivores, raising the question of the reservoir role of jackals for certain tick-borne pathogens. Except for *Haemaphysalis* ticks, another commonly reported tick on golden jackals from Asia is *Rhipicephalus haemaphysaloides*, a tick which prefers ungulates and known as vector of several viral and protozoan diseases [331]. Studies in golden jackals from arid regions (northern Africa and Middle East) demonstrated the predominant presence of ticks from the genus *Rhipicephalus*: *R. sanguineus* (*s.l*.), *R. turanicus* and *R. leporis*. Surprisingly, there are no reports of ticks on golden jackals in sub-Saharan Africa.

### Mites

Compared to foxes, jackals seem to be less affected by mange-causing mites (Table 7). So far, there is a single report of *Sarcoptes scabiei* in golden jackals, in Israel [287] and a single report of *Otodectes cynotis*, in Iran [295]. It is unclear if the scarcity of data regarding *Sarcoptes* is because of the low prevalence or because of the lack of studies and/or reports. Except sarcoptid mites, there are few records of *Demodex* in golden jackals, but its clinical significance is not known (Table 7).

### Fleas and lice

The diversity of fleas reported in golden jackals is relatively high, with at least seven species reported (Table 7), with the most common being *Pulex irritans*, *Ctenocephalides canis* and *C. felis*. Most of the reports of fleas in golden jackals originate in Russia and other former USSR countries and western and southern Asia. Surprisingly, there are no reports of fleas in golden jackals in Europe. The reports of lice in golden jackals are scarce with only three species occasionally reported (Table 7).

### Other arthropods

Although relatively common in most wild carnivores and domestic dogs (Mihalca, personal observation), there is only a single report of *Hippobosca longipennis* in golden jackals (Table 7). This species is an important vector for *Acanthocheilonema dracunculoides*, a filarioid widely distributed in canids across Africa [332]. However, this vector-borne nematode was never reported in golden jackals.

## Conclusions

This is the first comprehensive checklist summarizing the data on parasites of golden jackals. The large variety of parasites reported in golden jackals is caused by multiple factors, including their large geographical range, their extensive territorial mobility and wide food spectrum. Moreover, like in other carnivores, the predator behaviour of golden jackals is the cause of common records of pseudoparasites. Nevertheless, even in such cases, although these parasites do not infect jackals, they can be spread and can remain infective for their natural hosts. Considering that jackals share their habitats with domestic dogs and a wide variety of wild carnivores across their distribution range and the high similarity with canine parasites [333], the risk of interspecific transmission among canid species, and the continued spread of the species, is likely to be associated with future territorial expanding of different parasitic diseases. The vast majority of parasites recorded in golden jackals are shared with domestic dogs or even domestic cats. Other parasites of jackals can use a wide variety of other domestic species, including livestock, as intermediate hosts. Hence, jackals are an important source of infection for domestic animals and might be directly or indirectly responsible for economic losses. Probably the most important aspect regarding the parasites of golden jackal is the large number and common occurrence of zoonotic parasites. Among these, several are with high public health impact: *Leishmania*, *Echinococcus*, hookworms, *Toxocara*, and *Trichinella*. Our review brings overwhelming evidence on the importance of *Canis aureus* as wild reservoir of human parasites.
